# Retinal Vascular Fractal Dimension, Childhood IQ, and Cognitive Ability in Old Age: The Lothian Birth Cohort Study 1936

**DOI:** 10.1371/journal.pone.0121119

**Published:** 2015-03-27

**Authors:** Adele M. Taylor, Thomas J. MacGillivray, Ross D. Henderson, Lasma Ilzina, Baljean Dhillon, John M. Starr, Ian J. Deary

**Affiliations:** 1 Department of Psychology, University of Edinburgh, Edinburgh, United Kingdom; 2 Centre for Clinical Brain Sciences, University of Edinburgh, Edinburgh, United Kingdom; 3 Clinical Research Imaging Centre, University of Edinburgh, Edinburgh, United Kingdom; 4 Clinical Research Facility, University of Edinburgh, Edinburgh, United Kingdom; 5 Department of Ophthalmology, Princess Alexandra Eye Pavilion, Edinburgh, United Kingdom; 6 Centre for Cognitive Ageing and Cognitive Epidemiology, University of Edinburgh, Edinburgh, United Kingdom; 7 Alzheimer Scotland Dementia Research Centre, University of Edinburgh, Edinburgh, United Kingdom; University of Glasgow, UNITED KINGDOM

## Abstract

**Purpose:**

Cerebral microvascular disease is associated with dementia. Differences in the topography of the retinal vascular network may be a marker for cerebrovascular disease. The association between cerebral microvascular state and non-pathological cognitive ageing is less clear, particularly because studies are rarely able to adjust for pre-morbid cognitive ability level. We measured retinal vascular fractal dimension (*D*_*f*_) as a potential marker of cerebral microvascular disease. We examined the extent to which it contributes to differences in non-pathological cognitive ability in old age, after adjusting for childhood mental ability.

**Methods:**

Participants from the Lothian Birth Cohort 1936 Study (LBC1936) had cognitive ability assessments and retinal photographs taken of both eyes aged around 73 years (*n* = 648). IQ scores were available from childhood. Retinal vascular *D*_*f*_ was calculated with monofractal and multifractal analysis, performed on custom-written software. Multiple regression models were applied to determine associations between retinal vascular *D*_*f*_ and general cognitive ability (*g*), processing speed, and memory.

**Results:**

Only three out of 24 comparisons (two eyes × four *D*_*f*_ parameters × three cognitive measures) were found to be significant. This is little more than would be expected by chance. No single association was verified by an equivalent association in the contralateral eye.

**Conclusions:**

The results show little evidence that fractal measures of retinal vascular differences are associated with non-pathological cognitive ageing.

## Introduction

Cerebrovascular disease is a major cause of cognitive dysfunction, contributing to cognitive impairment in individuals with and without dementia [1,2]. No single set of cognitive disorders characterise cerebrovascular disease [3], and due to difficulties in viewing the cerebral small vessels in vivo, the true burden of cerebral microvascular disease on cognitive function is still poorly understood [4].

The homology of the retinal and cerebral microvasculatures has led researchers to investigate the potential of the retinal microvasculature as a marker of cerebral microvascular state [5]. The vascular network is designed for optimal hemodynamic and distributive functions [6,7] and variation in retinal vascular parameters, such as vessel calibre and branching geometry, may signify a deviation from this. It follows that these retinal changes are a potential marker of cerebrovascular abnormality and disease.

Retinal imaging allows non-invasive, *in vivo* assessment of the state of the vasculature [8], and is widely used to study systemic vascular disease [9–11]. Some clinical and population-based studies show that retinal microvascular abnormalities are associated with recognised signs of cerebral microvascular pathology, including cerebral microbleeds [12], white matter lesions [13,14], and brain infarcts [15,16]. Importantly, retinal signs are also related to ability in a range of cognitive domains [17–20], and with increased risk of cognitive impairment [21,22] and dementia [23]. Ding et al [24] provided a systematic review of this area. The most consistent associations have been found in studies investigating advanced retinal and cognitive abnormalities, such as those using qualitative measures of retinal abnormality, specifically severe retinopathy, and in those investigating associations with cognitive dysfunction and dementia rather than differences in ‘normal’ cognitive ability. Associations have been weaker and less consistent for less severe retinal abnormalities, quantitative retinal measures, and for non-impaired cognitive function. Combined with the inconsistency in sensitivity and robustness of cognitive measures used across the studies, as yet, there is little hard evidence that retinal vessel differences are associated with non-impaired cognitive ability in older age.

Recently, researchers have attempted to quantify retinal vascular changes using fractal analysis. This method summarises the global complexity of retinal vascular branching, combining information about vessel widths, vessel tortuosity, and branching geometry and density, to give a single dimensionless quantity: the fractal dimension (*D*_*f*_) [25]. Applied to retinal images, it has potential to be a highly sensitive gauge of microvascular disease. A suboptimal retinal vascular network increases energy costs and reduces efficiency of metabolic transport; microvascular health is maintained by both adequate vessel diameters and optimal branching architecture [26]. Low *D*_*f*_ values, which deviate from the idealised space-filling state of an “optimized” vascular branching network (*D*_*f*_ = 1.7) [27–30] have previously been associated with stroke [31], diabetic retinopathy [32], coronary heart disease mortality [26], cognitive dysfunction [22], and dementia [23]. However, it is not known if deviations from optimality are also related to differences in non-pathologic cognitive ageing. Furthermore, most studies have relied on monofractal analysis; a method which has generally achieved limited success [33]. Greater success has been reported by studies using multifractal techniques which characterise the retinal vasculature with a hierarchy of exponents rather than a single fractal dimension [34].

An important methodological point is that few studies to date have made adjustments for differences in prior cognitive ability when investigating the association between retinal vascular parameters and cognitive ability later in life. This is important because childhood cognitive ability accounts for a large proportion of variance in cognitive ability in old age [35]. There is mounting evidence from the field of cognitive epidemiology showing that differences in prior cognitive ability are associated with health, morbidity and mortality outcomes, including those related to cerebrovascular disease [36,37]. It is possible that previously found cross-sectional associations between retinal vascular measures and cognitive ability are confounded by prior cognitive ability. Shalev et al conducted one of the few studies which reported an association between non-pathological cognitive ability and retinal vascular measures. They found that wider retinal venular calibre measured at age 38 years was significantly associated with poorer cognitive ability measured in childhood, and in adulthood [20]. However, they did not adjust for childhood cognitive ability in the association between retinal venular calibre and adulthood cognitive ability, and as a result, the degree to which retinal vascular differences independently accounted for variance in age 38 cognitive ability was unclear. If retinal vascular differences are related to normal cognitive function, it is possible that their contribution is small in relation to that of childhood cognitive ability. Patton and colleagues found childhood IQ to account for almost 20% of the variance in cognitive ability assessed in old age, whilst retinal parameters accounted for 3.7% at the most [19]. The present study is rare in having a valid measure of childhood cognitive ability, retinal imaging data, and a comprehensive set of cognitive ability measures from old age.

In this study we investigated the extent to which retinal vascular fractal dimension (as a possible marker of cerebral microvascular disease) contributes to differences in non-pathological cognitive ability in old age, independently of childhood mental ability.

## Methods

Ethical permission for the Lothian Birth Cohort 1936 (LBC1936) study protocol was obtained from the Multi-Centre Research Ethics Committee for Scotland and from the Lothian Research Ethics Committee for Scotland. The research was carried out in compliance with the Helsinki Declaration. Written, informed consent was given by all participants.

### LBC1936 Sample

**Fig 1:**
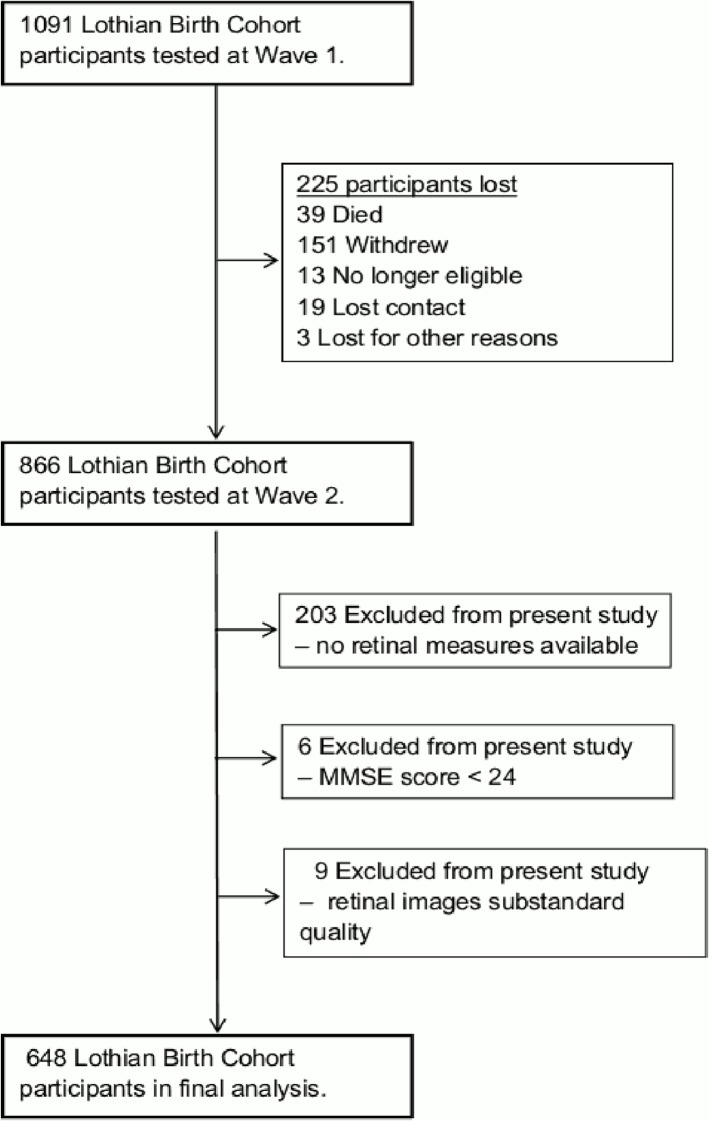
Flow diagram of exclusion factors associated with the study population.

Participants were a sub-sample of the LBC1936: a longitudinal study of cognitive ageing. This cohort comprises 1091 men and women, most of whom took part in the Scottish Mental Survey 1947 (SMS1947) [35,38]. Initial assessment took place between 2004 and 2007 when participants were about 70 years of age (M = 69.5, ± 0.8). The majority were living in the city of Edinburgh and surrounding Lothian area. A comprehensive description of recruitment and assessment procedures is available in an open-access protocol paper [39]. Data for the current study was mainly drawn from a second wave of the study (*n* = 866) [40]. Briefly, participants gave sociodemographic and medical history details during structured interviews, underwent repeat cognitive and physical testing, and completed a series of questionnaires including lifestyle and personality items, at about age 73 (M = 72.5, ± 0.7). Retinal photographs were taken at this time. Six hundred and sixty three participants had at least one image suitable for fractal analysis. Individuals with a Mini-Mental State Examination (MMSE) score < 24 were excluded (*n* = 6); this cut-off was used to exclude individuals with possible dementia. Participants with images of substandard quality for fractal analysis were also excluded (*n* = 9). A total of 648 remained for statistical analyses (see Fig. 1).

### Cognitive Ability

#### Moray House Test No. 12

The Moray House Test (MHT) No. 12 is a psychometric intelligence test which was validated at age 11 years against the Terman-Merrill revision of the Binet Scales [41,42]. It encompasses a range of items including reasoning, arithmetic, and spatial items, has a time-limit of 45 minutes, and a maximum score of 76. Participants first sat the MHT on 4th June 1947 as part of the SMS1947, along with almost all schoolchildren in Scotland aged about 11 years. MHT scores were corrected for age in days at time of testing and converted to an IQ-type scale for the full LBC1936 sample (mean = 100, SD = 15). This will be referred to as age 11 IQ. Of the 648 participants included in the analyses, 605 participants had completed the MHT at age 11.

#### Cognitive Domains

A general cognitive ability factor (*g*) was derived from principal components analysis (PCA) of six Wechsler Adult Intelligence Scale-III (WAIS-III) [43] subtests: Symbol Search, Digit Symbol Coding, Matrix Reasoning, Letter-Number Sequencing, and Block Design. Likewise, a processing speed factor was derived from scores on a set of speed measures: Symbol Search and Digit Symbol Coding from WAIS-III; and also measures of Simple and Four-Choice Reaction Time [44], and Inspection Time [45]. The same method was used to derive a memory factor from two WAIS-III subtests (Letter-Number Sequencing and Digit Span Backwards) and six Wechsler Memory Scale (WMS-III) subtests [46]: Logical Memory I Immediate Recall and II Delayed Recall, Spatial Span Forwards and Spatial Span Backwards, and Verbal Paired Associates I Immediate Recall and II Delayed Recall. The extraction of these factors was described previously [47,48].

### Additional Variables

Interview with a trained psychologist elicited self-reported presence or history of hypertension, cardiovascular disease, stroke, diabetes, and current smoking status (current smoker, ex-smoker, or non-smoker).

### Retinal Image Analysis

**Fig 2:**
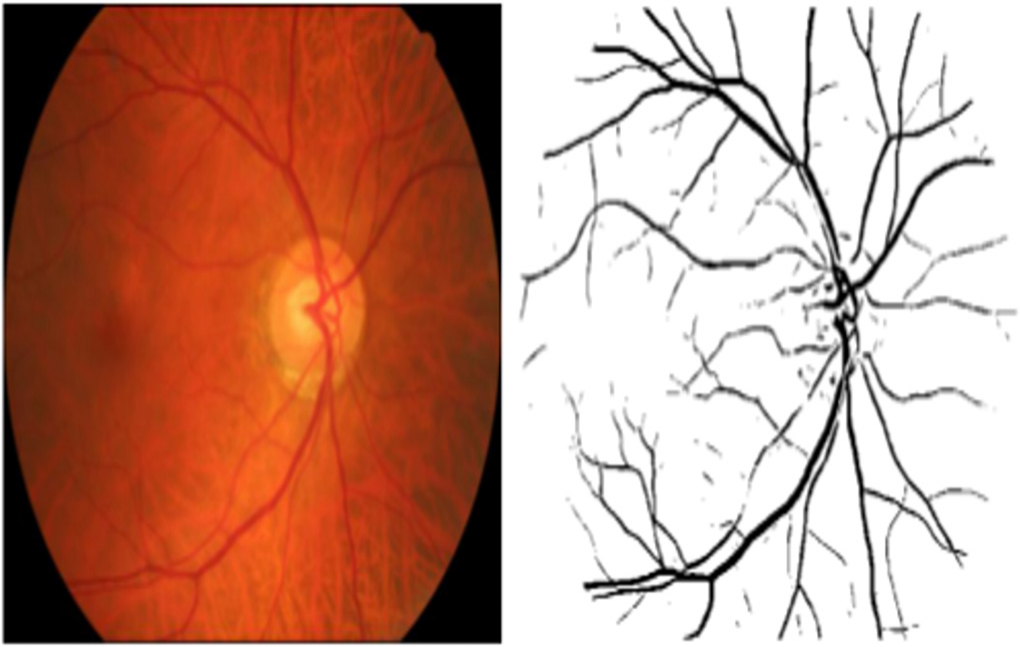
Colour retinal image acquired by fundus photography and retinal blood vessels segmented by computerized procedure.

Retinal images from both eyes were captured with a nonmydriatic camera at 45o FOV (CRDGi; Canon USA Inc., Lake Success, NY) and stored with 8 bits per colour plane, at 2048 × 3072 pixels and in TIFF format. Images were down-sampled to 685 x 584 pixels prior to processing and analysed by an expert retinal image analyst (TM) using custom software built in MATLAB (The MathWorks, Natwick, MA) [49]. Automatic segmentation of the retinal microvascular network (arterioles and venules) was performed using an algorithm described previously [50] which classifies each pixel of a fundus image as *vessel* or *non-vessel* to produce a segmented map of the retinal vasculature (see Fig. 2). Prior to fractal analysis computationally segmented images were inspected and obvious artefacts, such as areas of low contrast noise or “ring” artefacts caused by dust on the camera, were corrected or removed. Fractal analysis may be more sensitive to changes in vascular patterns when skeletons rather than images containing vessel width information are considered [51]. Therefore all images were skeletonised by iterative deletion of pixels [52]. Finally, monofractal and multifractal analysis was applied.

In the monofractal approach a single *D*_*f*_ (D_box_) was measured using box-counting: the vessel map or skeletonized version was overlaid with a series of boxes of increasing size *l*, and the number of boxes *N* containing at least one object pixel (representing part of the retinal vascular tree) was counted. The logarithm of *N* was plotted against the logarithm of *l*. D_box_ was the slope of the best fit straight line of the plotted points [53].

In the multifractal approach multiple fractal dimensions were measured at 1000 random points on the vessel maps and skeletonised images. The generalized sandbox method [34] was used to investigate the effect of scale on the *D*_*f*_. Multifractal dimensions *D*_*f*_ for real *f* are a more versatile parameter in describing geometrical properties. Had the retinal vessel tree been a simple monofractal, the *D*_*f*_ would have been constant for all scales, *f*. However, as expected the retinal tree displayed multifractal properties, giving different fractal dimensions as the scale was changed. We computed multifractal dimensions D_0_, D_1_, and D_2_ as these have previously been reported as likely sensitive markers of small vascular changes [34]. D_0_, D_1_ and D_2_ are commonly referred to as the capacity dimension, the entropy dimension and the correlation dimension, respectively. As we were unsure of the optimum one to use we investigated all three. Comparing the results enabled us to establish which was the most effective.

Implementation of this algorithm was trained and tested using a set of 20 images, manually segmented by 2 human observers as well as by the software. Fractal analysis of the manual segmentation performed by the ground truth observer was taken as the reference with which to compare fractal analysis of a second manual segmentation by a second observer and also the computational segmentation. A Bland-Altman approach [54] was used to assess the agreement between *D*_*f*_ measurements, with mean differences and coefficients of repeatability (CR) computed. These are summarized in Table 1.

**Table 1:** Mean Differences and Coefficient of Repeatability Between Retinal Vascular Fractal Dimension derived by Human Observer and Software Against Ground Truth.

Fractal Dimension	Segmentation Method Comparison		Mean difference	CR
D_box_	G	H	-0.02	0.04
	G	S	0.01	0.09
D_0_	G	H	0.02	0.05
	G	S	0.01	0.13
D_1_	G	H	0.01	0.05
	G	S	0.02	0.13
D_2_	G	H	0.00	0.06
	G	S	0.01	0.14

*D_box_ = monofractal dimension

D_0_, D_1_, D_2_ = multifractal dimension

^†^ G = ground truth: manual segmentation by first human observer

H = manual segmentation by second human observer

S = automated segmentation by software

### Statistical Analysis

All analyses were conducted using SPSS version 21 (IBM, NY, USA). There were moderate to strong, but far from perfect, correlations between left and right eye fractal dimensions (Pearson coefficients between 0.403–0.582); therefore, we conducted analyses on left and right eyes separately. Retinal vascular parameters were analysed as continuous (per 1 SD increase). Cognitive domain and individual subtest scores were normally distributed; we therefore used multiple linear regression with cognitive domains and subtest scores as dependent variables, and the retinal microvascular fractal dimensions as independent variables. Three models were fitted to the data with adjustments for potential confounding factors made in each. Age and sex were covariates in all three models. Age 11 IQ was included as an additional covariate in the second model. The third model adjusted for age, sex, age 11 IQ, hypertension, cardiovascular disease, diabetes, stroke, and current smoking status (non-smoker or ex-smoker vs. current smoker). A post hoc power analysis using pwr.r.test from the pwr package in R revealed that 648 subjects would provide 80% power to detect correlation coefficients of small effect size (.11; after Cohen) [55] with alpha set at. 05, two-tailed.

## Results

The mean of the differences between fractal dimensions for two observers and between the first (ground truth) observer and the computation segmentation were very similar in magnitude, though the CR was slightly larger for computational segmentation evaluated against first observer (see Table 1). However, the results indicated that the computational segmentation was within an acceptable level of performance for the identification of vessels (i.e. it was similar to someone repeating manual segmentation) and so provided suitable input to fractal analysis.

The baseline characteristics for the cohort are summarized in Table 2. Of the 648 participants (326 men, 50.3%) included in the analyses, seven participants did not have a general (*g*) cognition score, 32 did not have a processing speed score, 27 did not have a memory score, and 120 did not have the full range of *D*_*f*_ measurements. Mean cognitive domain scores and retinal vascular *D*_*f*_ values are summarized in Table 3. Greater *D*_*f*_ represents greater branching complexity. Greater *g*, processing speed, and memory domain scores represent better performance. The cognitive domain scores reported are standardised variables (mean = 0, SD = 1) for the whole sample.

**Table 2:** Baseline Characteristics of Participants with Retinal Images of Sufficient and Insufficient Quality for Fractal Analysis.

Characteristics	Image quality	
	Gradable* (*n* = 648)	Ungradeable^†^ (*n* = 9)
Age, years, mean (SD)	72.4 (0.71)	72.4 (0.74)
Male, n (%)	326 (50.3)	6 (66.7)
Age 11 IQ, mean (SD)	102 (14.0)	108 (9.80)
Hypertension, n (%)	313 (48.3)	4 (44.4)
Diabetes, n (%)	61 (9.4)	0 (0)
Current smoker, n (%)	52 (8.0)	2 (22.2)
Cardiovascular disease, mean (%)	177 (27.3)	1 (11.1)
Stroke, n (%)	39 (6.0)	0 (0)

* Participants with at least one retinal image of sufficient quality for fractal analysis

^†^ Participants with retinal images of insufficient quality for fractal analysis

**Table 3:** Descriptive Statistics of the Cognitive Domains and Fractal Dimensions of the Retinal Vascular Network.

	*n*	Mean	SD	Range
Cognitive Domain				
General (*g*) Cognitive Ability	641	0.04	0.99	-3.59–3.11
Processing Speed	616	0.08	0.94	-3.18–2.75
Memory	621	0.02	0.98	-3.82–2.21
Left Eye Fractal Dimension				
D_box_	530	1.42	0.02	1.36–1.48
D_0_	531	1.68	0.02	1.59–1.76
D_1_	529	1.64	0.03	1.55–1.72
D_2_	528	1.62	0.03	1.54–1.70
Right Eye Fractal Dimension				
D_box_	537	1.43	0.02	1.35–1.50
D_0_	538	1.68	0.02	1.60–1.77
D_1_	539	1.65	0.03	1.57–1.75
D_2_	538	1.63	0.03	1.55–1.73

Mean, standard deviation, and range of cognitive domain scores and retinal vascular fractal dimensions.

D_box_ = monofractal dimension

D_0_, D_1_, D_2_ = multifractal dimension

*N* varies due to incomplete range of fractal dimension measurements and/or missing subtest data.

Table 4 presents the associations between retinal microvascular *D*_*f*_ and ability in the three cognitive domains. Three of the 24 tests conducted using the basic model were significant at the conventional p value of <. 05. This is slightly more than one would expect by chance, but no association would survive Bonferroni type correction for multiple statistical testing. Full Bonferroni correction would not be appropriate in this instance given that the retinal measures are not independent (Pearson coefficients between 0.708–0.982) and neither were the cognitive measures (Pearson coefficients between 0.438–0.735). In addition to this, no associations were verified by an equivalent association in the opposite eye, regardless of there being a moderate correlation between left and right eye measures.

**Table 4:** Multiple Regression Models using Retinal Vascular Fractal Dimension to predict Ability in Three Cognitive Domains.

Cognitive Domain, Fractal Dimension, & Laterality of Eye			Model 1			Model 2			Model 3		
			*n*	β	*P*	*n*	β	*P*	*n*	β	*P*
*g*	D_box_	R	532	0.04	.322	502	0.03	.353	502	0.04	.250
		L	523	-0.03	.478	485	-0.03	.432	485	-0.03	.395
	D_0_	R	533	0.08	.063 ^†^	503	0.02	.604	503	0.03	.436
		L	524	0.000	.995	486	-0.01	.900	486	-0.01	.825
	D_1_	R	534	0.10	.021*	503	0.03	.404	503	0.04	.324
		L	522	0.01	.904	484	0.000	.993	484	-0.01	.749
	D_2_	R	533	0.09	.035*	502	0.04	.259	502	0.04	.244
		L	521	0.001	.976	483	-0.01	.812	483	-0.02	.608
Speed	D_box_	R	512	0.04	.385	484	0.03	.447	484	0.04	.315
		L	503	-0.08	.094 ^†^	467	-0.06	.195	467	-0.05	.257
	D_0_	R	513	0.07	.094 ^†^	485	0.04	.341	485	0.05	.250
		L	504	-0.04	.386	468	-0.02	.625	468	-0.02	.698
	D_1_	R	514	0.10	.028*	485	0.06	.179	485	0.06	.136
		L	502	-0.04	.429	466	-0.03	.473	466	-0.03	.452
	D_2_	R	513	0.09	.035*	484	0.07	.115	484	0.07	.092 ^†^
		L	502	-0.03	.583	466	-0.03	.506	466	-0.03	.466
Memory	D_box_	R	513	-0.03	.539	485	-0.02	.673	485	-0.01	.811
		L	506	-0.07	.142	470	-0.08	.050*	470	-0.08	.040*
	D_0_	R	514	0.02	.719	486	-0.02	.545	486	-0.02	.611
		L	506	-0.05	.232	470	-0.07	.084 ^†^	470	-0.07	.069 ^†^
	D_1_	R	515	0.02	.734	486	-0.03	.421	486	-0.03	.466
		L	505	-0.06	.175	469	-0.07	.053 ^†^	469	-0.08	.038*
	D_2_	R	514	0.000	.992	485	-0.03	.419	485	-0.03	.439
		L	505	-0.06	.188	469	-0.08	.041*	469	-0.08	.029*

Data are presented as standardised beta coefficients reflecting change in cognitive domain score associated with an increase of 1 *SD* unit in fractal dimension. Model 1 adjusted for age and sex; Model 2 adjusted for age, sex, and age 11 IQ; Model 3 adjusted for age, sex, age 11 IQ, hypertension, diabetes, cardiovascular history, stroke, current smoking status.

D_box_ = monofractal dimension; D_0_, D_1_, D_2_ = multifractal dimension.

*N* varies due to incomplete range of fractal dimension measurements and/or missing subtest data.

* Significant (*p* < 0.05).

^†^ Trend (*p* < 0.10).

The unadjusted significant associations were as follows. In the basic age- and sex- adjusted model right eye D_1_ was significantly associated with *g* (β = 0.10, *P* = 0.021) and processing speed (β = 0.10, *P* = 0.028). Similarly, right eye D_2_ was significantly associated with *g* (β = 0.09, *P* = 0.035) and processing speed (β = 0.09, *P* = 0.035). There was also a statistical trend with right eye D_0_ (*g*: β = 0.08, *P* = 0.063; processing speed: β = 0.07, *P* = 0.094). No association was found between monofractal or multifractal retinal vascular *D*_*f*_ and memory in this model. With the exception of a statistical trend between left eye D_box_ and processing speed (β = -0.08, *P* = 0.094), no association was found between monofractal dimension D_box_ and any cognitive domain.

IQ at age 11 was not associated with any of the retinal microvascular D_*f*_ measures at age 73 (Left D_box_: β = 0.007, *P* = 0.875; Left D_0_: β = 0.03, *P* = 0.491; Left D_1_: β = 0.000, *P* = 0.996; Left D_2_: β = -0.004, *P* = 0.931; Right D_box_: β = 0.01, *P* = 0.809; Right D_0_: β = 0.08, *P* = 0.063; Right D_1_: β = 0.07, *P* = 0.111; Right D_2_: β = 0.05, *P* = 0.236). The addition of age 11 IQ as a covariate in model 2 weakened the associations between D_0_, D_1_, and D_2_ with *g* and processing speed, the β coefficients were reduced by more than half in some cases, and in all cases significance was lost. The further addition of all cardiovascular risk factors as covariates in model 3 did not alter these associations significantly, though there was a statistical trend between right eye D_2_ and processing speed (β = 0.07, *P* = 0.092). Accounting for age 11 IQ in the association between retinal D_*f*_ and memory had the converse effect to that seen with the other domain scores. After adjustment for age 11 IQ, left eye D_*f*_ analysed using monofractal and multifractal methods significantly predicted memory (D_box_: β = -0.08, *P* = 0.050; D_2_: β = -0.08, *P* = 0.041), and there was a statistical trend with D_0_ (β = -0.07, *P* = 0.084) and D_1_ (β = -0.07, *P* = 0.053). Significance was maintained when all cardiovascular risk factors were accounted for in model 3 (D_box_: β = -0.08, *P* = 0.040; D_1_: B = -0.08, *P* < 0.038; D_2_: β = -0.08, *P* = 0.029), again there was a statistical trend with D_0_ (β = -0.07, *P* = 0.069).

The reversed direction of association indicates that increased branching complexity predicted poorer memory performance. However, again these associations were not corroborated by an equivalent finding when right eye retinal measures were tested and are therefore unlikely to be meaningful. Taken together, our results show no evidence that differences in retinal vascular fractal dimension contribute to cognitive ability in old age. We suggest that in our sample, differences in childhood ability, but not retinal vascular parameters, account for much of the variance in cognitive ability in later life.

S1 Table contains results from the analysis of individual cognitive subtests.

## Discussion

Microvascular disease is a major contributor to cognitive dysfunction in individuals with and without dementia. However, its role in non-pathologic cognitive function is unclear. The present study investigated cross-sectional associations between retinal vascular *D*_*f*_, a putative marker of cerebral microvascular health, and three domains of cognitive ability in a sample of older adults without cognitive impairment. After adjustment for childhood IQ and traditional cardiovascular risk factors, greater left eye monofractal and multifractal dimensions (D_box_, D_1_, and D_2_) were associated with poorer memory performance, however, neither left nor right eye fractal dimensions were associated with *g* or processing speed, and no association was verified by an equivalent result in the contralateral eye.

The data on retinal microvascular topography and non-pathological cognitive ageing have been limited, and direct comparison with previous studies is not possible due to various methodological differences in the research to date. The Lothian Birth Cohort 1921 (LBC1921) [19] study examined the associations between quantitative measures of suboptimal retinal vascular branching geometry and cognitive ability in 321 men and women aged either 83 or 84. After adjusting for childhood IQ, cardiovascular risk factors, and socio-demographic variables, deviation from the optimal retinal vascular bifurcation angle accounted for 2.6% of the variance in logical memory test scores of older adults, while deviation from the optimal retinal vascular branching coefficient accounted for 3.4% of the variance in a general cognitive ability (*g*) score, and 3.7% in verbal fluency test score. Similar results were found in sample of 1007 men and women from the Dunedin Multidisciplinary Health and Development study, a population based cohort followed from birth [20]. In this study, wider venular calibre was associated with lower total and delayed Rey auditory verbal learning scores, lower scores on several tests of executive function, and lower scores on all components of WAIS-IV. Our study results differ from these data in that no association was found between retinal vascular *D*_*f*_ and measures of *g* or processing speed, and retinal vascular *D*_*f*_ was only weakly associated with memory, though this was not confirmed by the contralateral eye and therefore unlikely to be meaningful. Some of this difference may be attributable to the differences in mean age of the cohorts. The LBC1921 cohort was aged between 83 and 84 years old, an age at which between-person variability in cognitive ability scores and retinal vascular state may be increased; however, fractal dimension was not measured in the LBC1921 study, and it is not possible to say if cognitive ability would have been associated with retinal vascular *D*_*f*_ in that sample. The Dunedin study cohort had a mean age of 38, and their results may be more consistent with the hypothesis that cognitive impairment is more greatly influenced by cerebral microvascular disease in younger compared to older adults [18]. Though, it is unlikely that much disease would be present in people of this age.

Qualitative measures of retinal vascular abnormality have also been associated with cognitive ability in older age. Results from the population-based Atherosclerosis Risk in Communities (ARIC) study showed that in a sample of 8734 persons, individuals with any retinopathy, microaneurysm, retinal haemorrhage or soft exudates, were 2.6 to 3.39 times more likely to score below mean on the delayed word recall test than those without [17]. Wong and colleagues also found an association between presence of retinopathy and low scores on the digit symbol substitution test, in both the ARIC study and in another sample of 2211 participants from the population-based Cardiovascular Health Study (CHS) In contrast to quantitative measures designed to identify subtle changes to the vasculature, qualitative measures are indicative of more severe states and even diseases of the retinal microvasculature, which are often associated with the breakdown of the blood-brain barrier. This difference in severity of retinal vascular pathology may partly explain why associations between cognitive ability and qualitative measures have been more consistent than those assessing less severe changes, such as narrowing of the vessels [18].

The relationship between ‘normal’ cognitive ageing and retinal vascular fractal dimension has not previously been investigated. The optimal retinal vascular *D*_*f*_ is said to be 1.7, and deviations from this have previously been associated with ageing [33], diabetic retinopathy [25] and death resulting from coronary heart disease (CHD) [26]. Cheung and colleagues recently found a significant association between reduced retinal vascular *D*_*f*_ and cognitive dysfunction. Individuals with the lowest *D*_*f*_ were between 1.03 and 2.82 times more likely to have cognitive dysfunction [22]. In a separate study the same authors found, that for every 1 SD unit decrease in *D*_*f*_, the likelihood of having Alzheimer’s Disease rose by 1.54 [23].

This study has several strengths. It makes a novel contribution to research investigating the relationship between parameters of the retinal microvasculature and cognitive function. To our knowledge, it is the first to examine the relationship between retinal vascular fractal dimension and non-pathologic cognitive ability, and the only one to do so using both monofractal and multifractal methods of analysis. It is important to note that generalized dimension spectrums for each image analysed, showed that the retinal vessel structures were all geometrical multifractals. Second, we were able to make statistical adjustment for childhood cognitive ability, a factor known to account for a large proportion of variance in later life cognitive ability [35, 56]. Third, cognitive domain scores were based on a broad range of neuropsychological tests, giving a robust measure of general (*g*) cognitive ability, processing speed, and memory. Finally, in contrast to previous studies [32,33] by analysing left and right eye retinal measures separately we were able to show that no association was substantiated by the same result using measurements from the contralateral eye (see S1–S3 Figs).

Limitations of the study should also be noted. Problems exist with the automated segmentation algorithm: the edge of the optic disk is sometimes wrongly detected as a vessel, incorrect detections result from the underlying choroidals, gaps appear in the middle of vessels if the central light reflex is bright, some vessel paths are broken due to poor image contrast and very small vessels are sometimes missed. All of these issues lead to a reduction in segmentation accuracy and fractal dimension of the retinal vasculature. Irrespectively, our images have very good agreement between the segmentations by two observers and so for fractal analysis the mean differences and CRs were small (see Table 1). When comparing fractal dimensions for computerised segmentations and ground truth (first human segmentation), mean differences were also small and CRs were an acceptable level. These results show that for a reasonable image quality, repeatability of fractal analysis performed on computerised segmentations is close to the repeatability found when two observers independently perform manual tracing. For large epidemiological studies, i.e. greater than fifty images, this would be at acceptable level in order to detect small changes.

Second, adjustment was not made for myopic refractive errors. Increasing myopia is associated with reduced retinal vascular fractal dimension, though this reduction is likely to be minimal except in cases of high myopia [57]. Additionally, myopia is less prevalent in older individuals, and therefore in the LBC participants myopia may not affect results to the same extent as a younger sample [58]. Differences in retinal vascular fractal dimension have also been found when comparing images from normal eyes to those from individuals diagnosed with diabetic retinopathy [25, 32]. Though presence of diabetes was not part of the exclusion criteria for the present study, no single image judged as ungradeable belonged to a diabetic individual, and adjustment for the presence of diabetes was made in the analyses. Third, the LBC1936 is a self-selecting sample, and as such it is likely to be healthier and more cognitively able than the general population [56]. It should be noted however, that though retinal vascular *D*_*f*_ does not appear to be associated with cognitive function in this sample at age 73, associations may be found as the sample ages and vascular pathology progresses. And though an association with cognitive ability was not found in this study, that is not to say that individual differences in retinal vascular *D*_*f*_ are not indicative of abnormality and disease of small vessels in general. Small vessel disease is a systemic disorder [2], and though small vessels of the brain may bear the burden of this disease in some individuals, in others, organs through the body will be most affected [4]. Further research is required to understand the full potential of fractal analysis as an early indicator of disease in the brain and body. Finally, retinal images were taken during only one wave of testing, restricting us to cross-sectional analyses. Access to retinal images taken at multiple time points would allow examination of these associations in relation to longitudinal change.

## Conclusion

Three out of the 24 comparisons were found to be significant. Although this is more than one would expect by chance, no association would survive Bonferroni correction for multiple statistical testing. Significant unadjusted associations were weakened and lost significance after covarying for IQ at age 11 and cardiovascular risk factors, and not one association was verified by an equivalent finding using measurements from the contralateral eye. We cautiously suggest that our results do not reject the null hypothesis, that differences in retinal vascular *D*_*f*_ are not associated with cognitive ability in old age. Further research is required to elucidate the relationship between parameters of the retinal microvascular network and non-pathological cognitive function.

## Supporting Information

S1 Fig Change in cognitive domain score associated with 1 *SD* unit increase in fractal dimension. Dbox = monofractal dimension. D0, D1, D2 = multifractal dimension. β significant at ± 0.08 (*p* < 0.05).
(PDF)

S2 Fig Change in cognitive subtest score associated with 1 *SD* increase in right eye fractal dimension. Dbox = monofractal dimension. D0, D1, D2 = multifractal dimension. β significant at ± 0.08 (*p* < 0.05). 1: Symbol search, 2: digit symbol coding, 3: matrix reasoning, 4:letter-number sequencing, 5: digit span backwards, 6: block design, 7: simple reaction time, 8: 4-choice reaction time, 9: inspection time, 10: spatial span forwards, 11: spatial span backwards, 12: verbal paired associates immediate recall, 13: verbal paired associates delayed recall, 14: logical memory immediate recall, 15: logical memory delayed recall.
(PDF)

S3 Fig Change in cognitive subtest score associated with 1 *SD* increase in left eye fractal dimension. Dbox = monofractal dimension. D0, D1, D2 = multifractal dimension. β significant at ± 0.08 (*p* < 0.05). 1: Symbol search, 2: digit symbol coding, 3: matrix reasoning, 4: letter-number sequencing, 5: digit span backwards, 6: block design, 7: simple reaction time, 8: 4-choice reaction time, 9: inspection time, 10: spatial span forwards, 11: spatial span backwards, 12: verbal paired associates immediate recall, 13: verbal paired associates delayed recall, 14: logical memory immediate recall, 15: logical memory delayed recall.
(PDF)

S1 Table Multiple Regression Models Using Retinal Vascular Fractal Dimension to predict Ability in Cognitive Subtests. *Note*. * p < 0.05; † trend *p* < 0.10. D_box_ = monofractal dimension; D_0_, D_1_, D_2_ = multifractal dimension; VPA = verbal paired associates; LM = logical memory. Standardised beta coefficients reflect change in cognitive subtest score associated with an increase of 1 *SD* unit in fractal dimension. Model 1 adjusted for age and sex; Model 2 adjusted for age, sex, and age 11 IQ; Model 3 adjusted for age, sex, age 11 IQ, hypertension, diabetes, cardiovascular history, stroke, current smoking status. *N* varies due to incomplete range of fractal dimension measurements and/or missing subtest data.
(PDF)
