# Supplementary material for: Retinal Vascular Fractal Dimension, Childhood IQ, and Cognitive Ability in Old Age: The Lothian Birth Cohort Study 1936
Source: PLoS One. 2015 Mar 27;10(3):e0121119. doi: 10.1371/journal.pone.0121119 (PMC4376388; doi:10.1371/journal.pone.0121119)
Supplement: S1 Fig — Dbox = monofractal dimension. D0, D1, D2 = multifractal dimension. β significant at ± 0.08 (p < 0.05). (PDF) [file pone.0121119.s001.pdf]

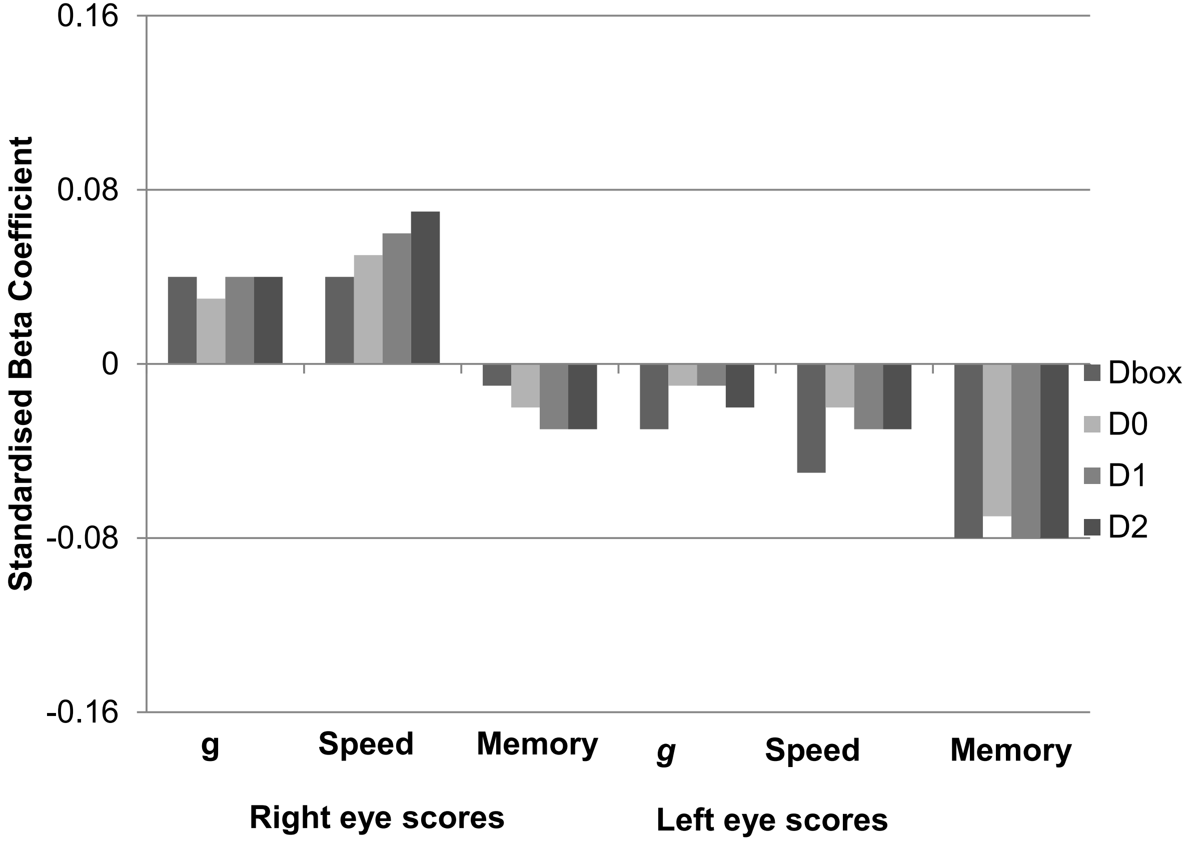

Supporting Information Figure S1. Change in cognitive domain score associated with an increase of 1 *SD* unit in right or left eye fractal dimension. Dbox = monofractal dimension. D0, D1, D2 = multifractal dimension. β significant at ± 0.08 (p < 0.05).
