# Supplementary material for: Retinal Vascular Fractal Dimension, Childhood IQ, and Cognitive Ability in Old Age: The Lothian Birth Cohort Study 1936
Source: PLoS One. 2015 Mar 27;10(3):e0121119. doi: 10.1371/journal.pone.0121119 (PMC4376388; doi:10.1371/journal.pone.0121119)
Supplement: S3 Fig — Dbox = monofractal dimension. D0, D1, D2 = multifractal dimension. β significant at ± 0.08 (p < 0.05). 1: Symbol search, 2: digit symbol coding, 3: matrix reasoning, 4: letter-number sequencing, 5: digit span backwards, 6: block design, 7: simple reaction time, 8: 4-choice reaction time, 9: inspection time, 10: spatial span forwards, 11: spatial span backwards, 12: verbal paired associates immediate recall, 13: verbal paired associates delayed recall, 14: logical memory immediate recall, 15: logical memory delayed recall. (PDF) [file pone.0121119.s003.pdf]

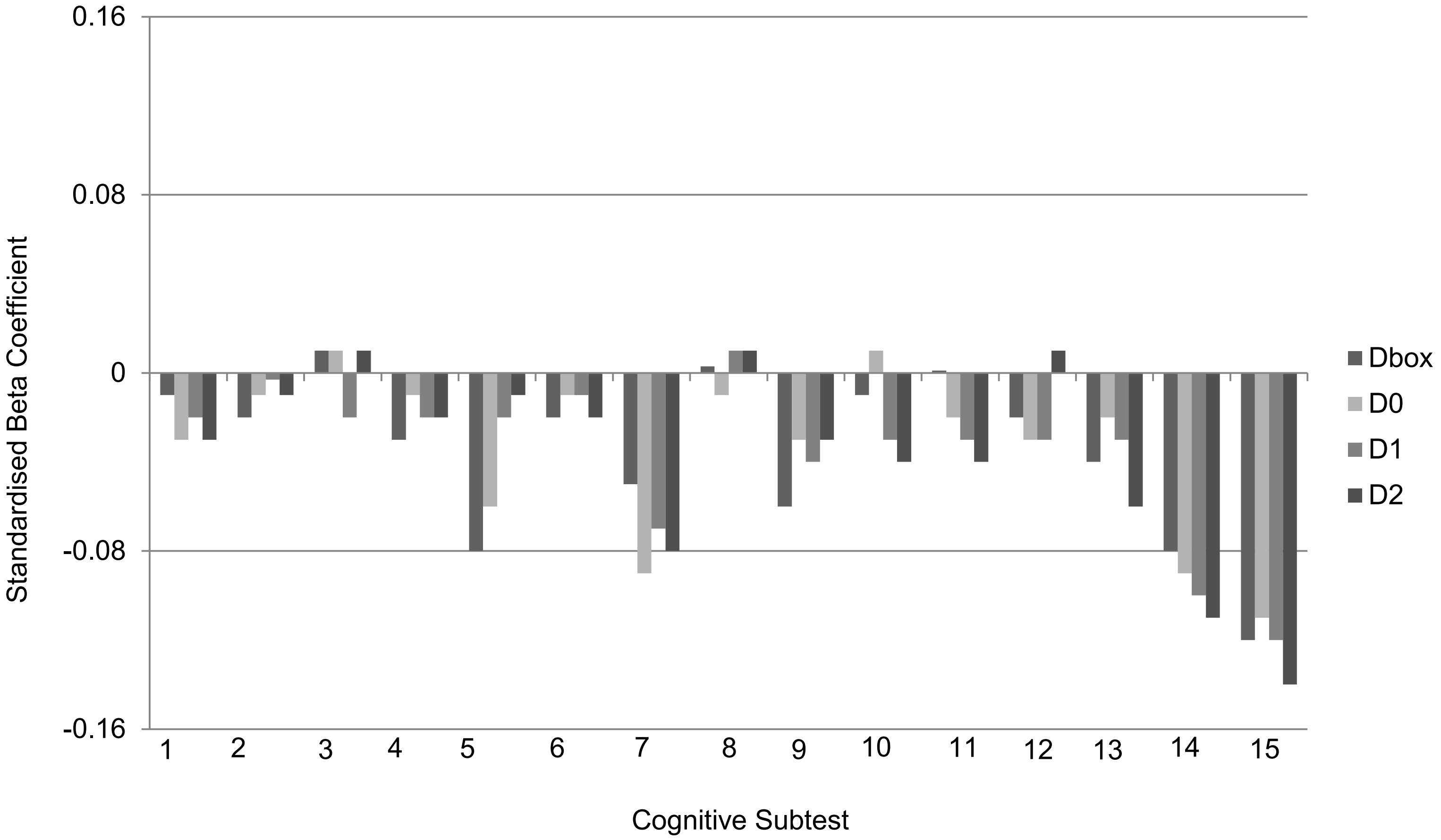

Supporting Information Figure S3. Change in cognitive subtest score associated with an increase of 1 *SD* unit in left eye fractal dimension. Dbox = monofractal dimension. D0, D1, D2 = multifractal dimension. β significant at ± 0.08 ($p < 0.05$). 1: Symbol search, 2: digit symbol coding, 3: matrix reasoning,

4: letter-number sequencing, 5: digit span backwards, 6: block design, 7: simple reaction time, 8: 4-choice reaction time, 9: inspection time, 10: spatial span forwards, 11: spatial span backwards, 12: verbal paired associates immediate recall, 13: verbal paired associates delayed recall, 14: logical memory immediate recall, 15: logical memory delayed recall.
