# Supplementary material for: Retinal Vascular Fractal Dimension, Childhood IQ, and Cognitive Ability in Old Age: The Lothian Birth Cohort Study 1936
Source: PLoS One. 2015 Mar 27;10(3):e0121119. doi: 10.1371/journal.pone.0121119 (PMC4376388; doi:10.1371/journal.pone.0121119)
Supplement: S1 Table — Note. * p < 0.05; † trend p < 0.10. Dbox = monofractal dimension; D0, D1, D2 = multifractal dimension; VPA = verbal paired associates; LM = logical memory. Standardised beta coefficients reflect change in cognitive subtest score associated with an increase of 1 SD unit in fractal dimension. Model 1 adjusted for age and sex; Model 2 adjusted for age, sex, and age 11 IQ; Model 3 adjusted for age, sex, age 11 IQ, hypertension, diabetes, cardiovascular history, stroke, current smoking status. N varies due to incomplete range of fractal dimension measurements and/or missing subtest data. (PDF) [file pone.0121119.s004.pdf]

Supporting Information Table S1. Multiple Regression Models Using Retinal Vascular Fractal Dimension to predict Ability in Cognitive Subtests.

**Cognitive Subtest**	**Fractal Dimension**	**Laterality of Eye**	**Model 1**			**Model 2**			**Model 3**		
			N	**β**	***P***	***N***	**β**	***P***	***N***	**β**	***P***
Symbol Search	D_{box}	R	537	0.06	.174	506	0.07	**.096 †**	506	0.08	**.051 †**
		L	530	-0.02	.689	491	-0.01	.799	491	-0.01	.741
	D_0	R	538	0.10	**.016***	507	0.08	**.058 †**	507	0.09	**.032***
		L	531	-0.03	.439	492	-0.03	.443	492	-0.03	.428
	D_1	R	539	0.13	**.003***	507	0.10	**.014***	507	0.11	**.009***
		L	529	-0.02	.691	490	-0.01	.765	490	-0.02	.571
	D_2	R	538	0.13	**.003***	506	0.11	**.006***	506	0.12	**.004***
		L	528	-0.02	.636	489	-0.02	.642	489	-0.03	.486
Digit Symbol Substitution	D_{box}	R	537	0.04	.424	506	0.04	.387	506	0.05	.267
		L	530	-0.02	.648	491	-0.03	.482	491	-0.02	.551
	D_0	R	538	0.05	.198	507	0.02	.565	507	0.03	.448
		L	531	-0.002	.957	492	-0.01	.737	492	-0.01	.763
	D_1	R	539	0.09	**.043***	507	0.04	.277	507	0.05	.222
		L	529	0.01	.749	490	0.003	.949	490	-0.003	.944
	D_2	R	538	0.09	**.037***	506	0.06	.130	506	0.06	.110
		L	528	0.01	.812	489	-0.004	.920	489	-0.01	.851
Matrix Reasoning	D_{box}	R	537	0.02	.622	506	0.02	.649	506	0.02	.582
		L	529	0.01	.848	490	0.01	.754	490	0.01	.804
	D_0	R	538	0.03	.487	507	-0.01	.787	507	-0.01	.866
		L	530	0.02	.670	491	0.01	.800	491	0.01	.832
	D_1	R	539	0.04	.321	507	-0.01	.852	507	-0.01	.889
		L	528	0.003	.946	489	-0.01	.857	489	-0.02	.705

	D_2	R	538	0.03	.436	506	-0.01	.900	506	-0.01	.892
		L	527	-0.002	.968	488	-0.01	.735	491	0.01	.832
Letter-Number Sequencing	D_{box}	R	536	-0.01	.831	505	-0.01	.872	505	-0.003	.934
		L	529	-0.02	.637	490	-0.03	.459	490	-0.03	.447
	D_0	R	537	0.05	.295	506	0.004	.912	506	0.01	.774
		L	530	0.01	.803	491	-0.01	.905	491	-0.01	.906
	D_1	R	538	0.06	.199	506	0.01	.750	506	0.02	.676
		L	528	-0.001	.988	489	-0.02	.669	489	-0.02	.617
	D_2	R	537	0.05	.250	505	0.02	.640	505	0.02	.627
		L	527	0.01	.893	488	-0.02	.718	488	-0.02	.691
Digit Span Backwards	D_{box}	R	537	-0.03	.551	506	-0.03	.416	506	-0.03	.419
		L	530	-0.06	.150	491	-0.08	.059	491	-0.08	**.052 †**
	D_0	R	538	0.01	.809	507	-0.03	.420	507	-0.04	.400
		L	531	-0.03	.437	492	-0.05	.255	492	-0.06	.192
	D_1	R	539	0.02	.629	507	-0.03	.430	507	-0.03	.403
		L	529	0.001	.988	490	-0.01	.777	490	-0.02	670
	D_2	R	538	0.01	.741	506	-0.03	.542	506	-0.03	.508
		L	528	0.01	.834	489	-0.01	.868	489	-0.01	.754
Block Design	D_{box}	R	536	0.06	.181	505	0.04	.278	505	0.05	.202
		L	529	-0.01	.842	490	-0.02	.642	490	-0.02	.567
	D_0	R	537	0.06	.153	506	0.01	.818	506	0.02	.599
		L	530	0.02	.735	491	-0.002	.962	491	-0.01	.821
	D_1	R	538	0.08	**.079 †**	506	0.02	.656	506	0.02	.546
		L	528	0.01	.741	489	0.01	.830	489	-0.01	.857

	D_2	R	537	0.06	.157	505	0.02	.603	505	0.02	.593
		L	527	0.002	.972	488	-0.004	.922	488	-0.02	.663
4-Choice Reaction Time	D_{box}	R	537	-0.03	.576	506	-0.04	.379	506	-0.05	.308
		L	530	-0.01	.754	491	0.01	.785	491	0.003	.953
	D_0	R	538	-0.06	.158	507	-0.05	.302	507	-0.05	.278
		L	531	-0.03	.540	492	-0.01	.874	492	-0.01	.805
	D_1	R	539	-0.07	.134	507	-0.05	.295	507	-0.05	.278
		L	529	-0.01	.778	490	0.01	.769	490	0.01	.809
	D_2	R	538	-0.05	.255	506	-0.04	.360	506	-0.04	.343
		L	528	-0.01	.786	489	0.02	.733	489	0.01	.789
Simple Reaction Time	D_{box}	R	537	-0.02	.587	506	-0.02	.701	506	-0.02	.719
		L	530	-0.05	.282	491	-0.05	.306	491	-0.05	.274
	D_0	R	538	-0.02	.680	507	0.01	.884	507	0.01	.828
		L	531	-0.08	**.073 †**	492	-0.09	**.052 †**	492	-0.09	**.048***
	D_1	R	539	-0.01	.841	507	0.01	.782	507	0.02	.732
		L	529	-0.06	.203	490	-0.07	.127	490	-0.07	.106
	D_2	R	538	0.01	.837	506	0.02	.611	506	0.02	.587
		L	528	-0.06	.175	489	-0.07	.100	489	-0.08	**.087 †**
Inspection Time	D_{box}	R	525	-0.02	.655	495	-0.04	.390	495	-0.03	.481
		L	517	-0.05	.281	479	-0.06	.186	479	-0.06	.223
	D_0	R	526	0.02	.592	496	-0.02	.600	496	-0.02	.686
		L	518	-0.02	.610	480	-0.03	.459	480	-0.03	.523
	D_1	R	527	0.01	.756	496	-0.03	.577	496	-0.02	.640
		L	516	-0.02	.650	478	-0.03	.448	478	-0.04	.443
	D_2	R	526	0.01	.742	495	-0.01	.799	495	-0.01	.847
		L	516	-0.01	.844	478	-0.03	.548	478	-0.03	.525

Spatial Span Forwards	D_{box}	R	536	0.01	.858	505	0.004	.922	505	0.01	.836
		L	529	0.004	.922	490	-0.01	.890	490	-0.01	.852
	D_0	R	537	0.01	.838	506	-0.02	.639	506	-0.01	.759
		L	530	0.004	.918	491	0.01	.913	491	0.01	.904
	D_1	R	538	0.02	.603	506	-0.01	.765	506	-0.01	.852
		L	528	-0.01	.900	489	-0.02	.687	489	-0.03	.506
	D_2	R	537	0.02	.647	505	-0.004	.934	505	-0.001	.975
		L	527	-0.01	.744	488	-0.03	.464	488	-0.04	.357
Spatial Span Backwards	D_{box}	R	534	0.05	.266	504	0.05	.254	504	0.05	.224
		L	527	0.000	.995	489	0.01	.868	489	0.001	.984
	D_0	R	535	0.04	.333	505	0.01	.761	505	0.02	.608
		L	528	-0.02	.658	490	-0.02	.699	490	-0.02	.701
	D_1	R	536	0.07	**.090 †**	505	0.03	.439	505	0.04	.357
		L	526	-0.02	.599	488	-0.03	.575	488	-0.03	.451
	D_2	R	535	0.06	.140	504	0.04	.401	504	0.04	.365
		L	525	-0.03	.567	487	-0.03	.498	487	-0.04	.415
Verbal Paired Associates	D_{box}	R	526	0.03	.533	497	0.03	.457	497	0.04	.350
		L	516	-0.02	.675	479	-0.02	.716	479	-0.02	.683
	D_0	R	527	0.04	.362	498	0.01	.743	498	0.02	.681
		L	517	-0.03	.533	480	-0.03	.503	480	-0.03	.530
	D_1	R	528	0.01	.914	498	-0.02	.657	498	-0.02	.713
		L	515	-0.02	.610	478	-0.03	.513	478	-0.03	.498
	D_2	R	527	-0.02	.706	497	-0.03	.459	497	-0.03	.487
		L	514	-0.02	.595	477	-0.03	.481	477	-0.03	.457
VPA Delayed Recall	D_{box}	R	525	0.03	.575	496	0.03	.489	496	0.04	.353

		L	516	-0.05	.261	479	-0.04	.348	479	-0.04	.309
	D_0	R	526	0.07	.101	497	0.05	.277	497	0.05	.220
		L	517	-0.02	.612	480	-0.02	.662	480	-0.02	.726
	D_1	R	527	0.02	.590	497	0.000	1.000	497	0.01	.897
		L	515	-0.04	.380	478	-0.03	.493	478	-0.03	.455
	D_2	R	526	0.002	.966	496	-0.01	.736	496	-0.01	.802
		L	514	-0.07	.136	477	-0.06	.170	477	-0.06	.149
Logical Memory	D_{box}	R	535	-0.05	.281	504	-0.05	.244	504	-0.05	.249
		L	528	-0.08	**.062 †**	489	-0.08	**.048***	489	-0.08	**.045***
	D_0	R	536	-0.01	.775	505	-0.05	.206	505	-0.05	.183
		L	529	-0.07	.101	490	-0.08	**.043***	490	-0.09	**.034***
	D_1	R	537	-0.001	.986	505	-0.04	.276	505	-0.05	.254
		L	527	-0.10	**.028***	488	-0.09	**.020***	488	-0.10	**.018***
	D_2	R	536	-0.02	.705	504	-0.05	.229	504	-0.05	.218
		L	526	-0.10	**.022***	487	-0.10	**.013***	487	-0.11	**.011***
LM Delayed Recall	D_{box}	R	535	-0.04	.428	504	-0.03	.448	504	-0.03	.492
		L	528	-0.11	**.015***	489	-0.12	**.005***	489	-0.12	**.003***
	D_0	R	536	-0.004	.935	505	-0.03	.426	505	-0.03	.417
		L	529	-0.08	**.059 †**	490	-0.10	**.016***	490	-0.11	.010
	D_1	R	537	0.01	.865	505	-0.02	.572	505	-0.03	.547
		L	527	-0.10	**.021***	488	-0.11	**.009***	488	-0.12	**.004***
	D_2	R	536	-0.003	.950	504	-0.03	.546	504	-0.03	.509
		L	526	-0.11	**.009***	487	-0.13	**.002***	487	-0.14	**.001***

Note. * $p < 0.05$; † trend $p < 0.10$. D_{box} = monofractal dimension; D_0, D_1, D_2 = multifractal dimension; VPA = verbal paired associates; LM = logical memory. Standardised beta coefficients reflect change in cognitive subtest score associated with an increase of 1 *SD* unit in fractal dimension. Model 1 adjusted for

age and sex; Model 2 adjusted for age, sex, and age 11 IQ; Model 3 adjusted for age, sex, age 11 IQ, hypertension, diabetes, cardiovascular history, stroke, current smoking status. *N* varies due to incomplete range of fractal dimension measurements and/or missing subtest data.
